# A Review of Knowledge, Belief and Practice Regarding Osteoporosis among Adolescents and Young Adults

**DOI:** 10.3390/ijerph15081727

**Published:** 2018-08-12

**Authors:** Chin Yi Chan, Norazlina Mohamed, Soelaiman Ima-Nirwana, Kok-Yong Chin

**Affiliations:** Department of Pharmacology, Faculty of Medicine, Universiti Kebangsaan Malaysia, Jalan Yaacob Latif, Bandar Tun Razak, Cheras 56000, Kuala Lumpur, Malaysia; chanchinyi94@gmail.com (C.Y.C.); azlina@ppukm.ukm.edu.my (N.M.); imasoel@ppukm.ukm.edu.my (S.I.-N.)

**Keywords:** knowledge, beliefs, behaviours, diet, physical activity, adolescents, teenager, young adults, students, osteoporosis, bone health

## Abstract

Osteoporosis is a major public health problem affecting millions of people worldwide. Increasing knowledge, correcting health belief and promoting osteoprotective practices are effective measures for building and maintaining strong bone throughout ones’ life-span. This review aims to summarize the contemporary evidence on the knowledge, beliefs and practice of adolescents and young adults on bone health. We performed literature searches using the PubMed and Scopus databases to identify original studies from 2008 to May 2018 using the search terms “(knowledge OR beliefs OR attitude OR practice OR behaviours OR physical activity OR exercise OR diet OR nutrition) AND (young OR youth OR adolescents OR children OR young adults OR students OR teenager) AND (osteoporosis OR bone health)”. Of the 3206 articles found, 34 met the inclusion criteria. Studies showed that most adolescents and young adults had poor knowledge and expressed disinterest in osteoporosis. They believed that other diseases were more serious than osteoporosis, contributing to low perceived susceptibility and seriousness towards this disease. Popular media emerged as a platform to obtain information regarding osteoporosis. The lack of knowledge and misconceptions about osteoporosis led to poor osteoprotective practices. As a conclusion, the current evidence revealed a lack of awareness about osteoporosis among adolescents and young adults. Educational interventions may be useful to improve the awareness of osteoporosis among this population.

## 1. Introduction

Osteoporosis is metabolic skeletal disease, in which the bone becomes porous, brittle, and more susceptible to fractures [1]. It is an increasingly important health problem as the elderly population expands rapidly worldwide [2]. An epidemiological study estimated that 9–38% women and 1–8% men >50 years from industrialized countries suffered from osteoporosis [3]. The skeletal system is in a state of constant regeneration, through the process of bone formation and resorption. Bone formation supersedes bone resorption during growth while the reverse occurs among the middle-aged and the elderly [4]. An imbalance in bone remodelling skewing towards resorption will lead to bone loss and eventually osteoporosis.

Osteoporosis is preventable by optimizing peak bone mass during skeletal growth, preserving bone mass during adulthood, and minimizing bone loss during old age [5]. The skeletal system undergoes rapid development between early childhood and late adolescence [6]. The greatest accrual of bone mineral density happens during adolescence, representing about 60% of the bone growth in a life time [6,7,8]. Up to 90 percent of peak bone mass is acquired by the age 18 in girls and 20 in boys, which makes youth the best time to invest in ones’ bone health [9]. Since the bone density decreases with age, acquisition of peak bone mass during the first three decades and the following retention of bone through middle-age are essential determinants for reducing the risk of osteoporosis [10].

Understanding the factors that encourage osteoporosis preventive behaviours is important for the prevention of this disease at the population level. The reasons for engaging in osteoporosis preventive behaviours are complex because they are influenced by personal and social factors [11]. A study showed that social capital indirectly affected calcium intake through social support, self-efficacy for calcium intake and self-efficacy for exercise. Self-efficacy directly affected calcium intake and exercise among young adults, while self-efficacy, social support and participation in sports teams were strongly associated with bone health in adolescent girls [12]. Besides, knowledge of osteoporosis was reported to influence calcium intake and exercise indirectly through self-efficacy among students [13]. Another study suggested that identifying the barriers to calcium supplement could be one of the strategies to increase their calcium intake and reduce osteoporosis risk [14]. The study found that the leading barrier for young adults to consume calcium supplements was the lack of knowledge about the importance of increasing calcium intake, the lack of motivation to start supplements, and the belief that their dietary calcium intake alone was sufficient.

It is important to understand the knowledge, belief and practice of adolescents and young adults towards osteoporosis so that strategies to optimize their peak bone mass can be devised [15]. In addition, with proper knowledge, they can also improve the bone health of their family as they are the caretakers of senior members in their family. The current review aimed to provide a contemporary view of the knowledge, belief and practice among younger populations regarding osteoporosis. Evidence from the latest decade was summarized to update the readers on the latest findings in this field.

## 2. Materials and Methods

A literature search was performed from 15 April 2018 to 15 May 2018 on the PubMed and Scopus databases using keywords “(knowledge OR beliefs OR attitude OR practice OR behaviours OR physical activity OR exercise OR diet OR nutrition) AND (young OR youth OR adolescents OR children OR young adults OR students OR teenager) AND (osteoporosis OR bone health)”. All original research articles published between 2008 and May 2018 were considered to provide an up-to-date view on knowledge, attitude and practice of youths regarding bone health/osteoporosis. In this review, the definition of adolescents and young adults of World Health Organization was adopted, whereby adolescents are any person between the age of 10 and 19 years, whereas young adults are any person between 20–36 years [16].

**Figure 1:**
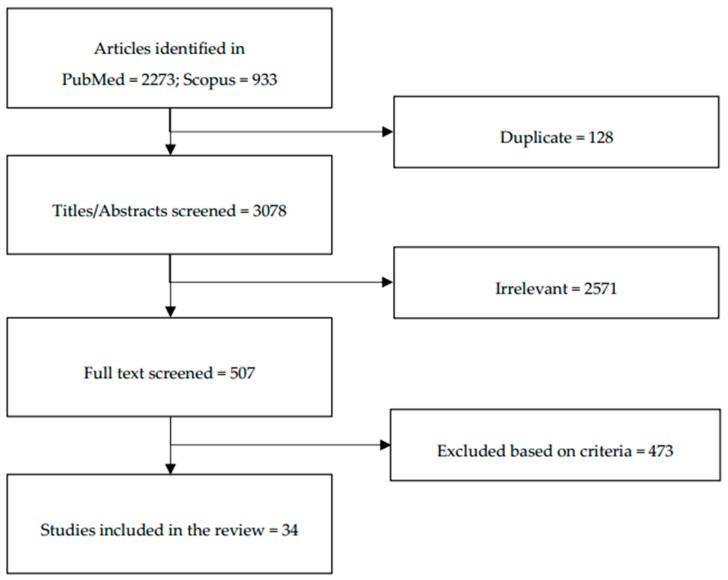
Flow diagram of the stepwise selection of relevant studies.

Our literature search identified 3206 articles (2273 from PubMed and 933 from Scopus). After removing duplicated articles (n = 128), each title and abstract for potential inclusion was screened. Studies involving participants aged ≥40 years and/or did not examine knowledge, attitude or practice regarding bone health/osteoporosis as the primary objective were excluded. Only original research articles written in English or Mandarin, with the main objective to study the knowledge, awareness, beliefs and practices among adolescents or young adults regarding bone health/osteoporosis, were included in this review. The full text of eligible articles was examined. Two reviewers decided articles to be included in the review. Any discrepancies between two reviewers were resolved by discussion. Data extraction on authors (year), subjects’ characteristics, study design, major findings were performed by the same authors. A total of 34 articles which met the criteria and provided sufficient information for data extraction and were included in this review (Figure 1).

## 3. Results

Thirty-four studies evaluating knowledge, attitude and practice regarding bone health/osteoporosis among adolescents and young adults were included in this review. The studies were performed in various countries and regions. The majority of the studies are from Saudi Arabia [15,17,18,19], Sri Lanka [20], Canada [21,22,23,24], Thailand [25,26] and South Korea [27,28,29,30], Jordan [31,32], Malaysia [33,34], China [35,36] and the rest are from United States [35], Nigeria [4], Taiwan [37], Syria [38], India [39], Poland [40], Denmark [41], U.K. [42], Iran [43], Pakistan [44], Columbia [45], the Basque region (Spain) [46], Japan [47] and Australia [48]. Most of the studies included in this review used either self-designed or validated questionnaire to assess the knowledge, attitude and practice among subjects regarding bone health/osteoporosis.

### 3.1. Knowledge Regarding Osteoporosis Among Adolescents and Young Adults

Out of the thirty-four studies, sixteen studies evaluated knowledge regarding osteoporosis. Only two studies [32,37] involved adolescents in their studies while others included young adult population. Background of the subjects varied among the studies; most of them were students from college [21,37], high school [32,37], polytechnic [4] or university [17,20,31,33,35,38,39,43,44,45]. The fields of study were medical and non-medical. Some of them were from the general population.

The knowledge level of the participants can be divided into good and poor. Four studies reported good knowledge levels among the younger generations regarding osteoporosis [31,33,37,39]. The studies recruited both male and female participants, consisting of university students [31,33,39] and a mixture of high school and college students [37]. Several factors contributed to the high level of osteoporosis knowledge among these studies. The subjects of the study of Amre et al. were nursing students. Integration of the preventive knowledge on osteoporosis into the learning and practice of the curriculum enhanced their understanding on this aspect of the disease. In comparison, they did not score well on the knowledge of pathophysiology because they did not apply this information [31]. Greater parental supervision on the bone health among Asian younger populations also contributed to a higher knowledge level on bone health [37]. Another multiracial study in Malaysia suggested that the ethnic group (Chinese) more susceptible to osteoporosis was more knowledgeable on the disease compared to the others (Malays and Indians) [33]. The students generally relied on teachers and textbooks to learn about osteoporosis [39]. These studies showed that the level of knowledge among younger generations depends on both formal education at school and parental supervision at home.

Six studies indicated that the adolescents or young adults had poor knowledge about osteoporosis [4,20,38,43,44]. From these studies, five studies [20,38,43,44] involved students from medical or health related disciplines and only one study was carried out among polytechnic students [4]. This is alarming because these students, who could be involved in the treatment and management of osteoporosis in the future, were not proficient on bone health. Gammage et al. recommended that these future health professionals should increase their knowledge on modifiable and non-modifiable risk factors of osteoporosis because most of the subjects failed to identify specific exercise related to bone health, genetic and medical conditions related to osteoporosis [21]. Part of this negligence also stemmed from the perception of the students that osteoporosis is a disease of the old age [4]. However, without adequate knowledge, the students, especially the females, would not be able to determine their own risk of developing the disease and change their health-related beliefs and behaviours [49].

Furthermore, some studies highlighted a difference in osteoporosis knowledge based on sex [17], study discipline [35] and country [45]. Alamri et al. demonstrated that female respondents were more knowledgeable compared to males, probably due to the belief that osteoporosis was a female disease [17]. A comparison study between US and Chinese university students showed that US students were more knowledgeable about osteoporosis. However, both groups were unable to identify the recommended daily calcium intake for adults [35]. Nguyen and Wang found that among health science programs, the level of education on osteoporosis differ significantly according to programs and levels of study of the students [45]. Thus, some students may not be equipped with sufficient knowledge to manage osteoporosis patients in the future.

The sources of bone health knowledge were discussed in some studies [15,25,32]. Three studies coherently indicated that the main source of knowledge regarding osteoporosis among teenagers was the television [15,25,32]. One study by Puttapitakpong et al. indicated that the internet was the second most popular source of information on bone health for teenagers [25]. This highlight the importance of media in educating adolescents and young adults about osteoporosis. The health care providers should also involve actively in osteoporosis awareness campaign through suitable channels.

A summary of the literature on the knowledge regarding osteoporosis among adolescents and young adults is listed in Table 1.

### 3.2. Beliefs Regarding Osteoporosis among Adolescents and Young Adults

Beliefs regarding osteoporosis are also summarized in this review. The number of studies reporting high [17,21,22,35] and low [20,33,37,38,44] individual perceived susceptibility of suffering from osteoporosis was quite similar. Sex differences in beliefs regarding osteoporosis was also noted in some studies [17,21,22,35]. Three studies revealed that young women perceived higher susceptibility regarding osteoporosis than young men [21,22,35], and only one study found the inverse [17]. This could be because women are known to be more likely to develop osteoporosis [21]. Despite that, young women showed significant lower exercise efficacy as compared to men. The researchers predicted that this might be due to factors such as fewer previous experiences, less social support, and more injuries related to physical activity, especially during childhood and adolescence. A study by Shanti et al. revealed no significant differences in perceived seriousness and health motivation scores across age and gender groups [22].

Some studies reported low perceived susceptibility towards osteoporosis among adolescents or young adults [20,33,37,38,44]. Among the five studies, four involved students with medical or health related background [20,33,38,44], showing that perceived susceptibility to osteoporosis was unsatisfactory even among those with basic medical knowledge. Chen et al. suggested that age might play an important factor in the osteoporosis beliefs, especially among Asian adolescents who tend to stay with their family. They demonstrated that, in comparison with young adults, adolescents did not believe they would suffer from osteoporosis, probably due to their young age. In contrast, they obtained higher scores in seriousness and prevention of osteoporosis, probably because they lived with seniors with osteoporosis at home [37]. This was supported by another study showing that young females who had their mother with history of low trauma fracture were significantly more likely to perceive osteoporosis as a serious disease [38]. A study found that younger generations had lower perceived seriousness of osteoporosis compared to cancer and diabetes [33]. The researchers mentioned that this might be due to the misconceptions that osteoporosis is an inevitable part of ageing and it is not a lethal or serious disease [33]. Most people also considered other diseases, such as heart diseases, HIV and diabetes pose more serious health consequences than osteoporosis [33].

In some studies, despite the high perceived seriousness of osteoporosis, perceived barriers to osteoprotective behaviours remained high [23,35,38]. Among the barriers to meet the daily recommendation of calcium intake were high cost and inconvenience of milk products (packaging), as well as negative practices of dairy farmers (hormones were given to cows to encourage milk production) [23]. Cultural differences, as demonstrated between US and Chinese university students, also contributed to discrepancies between dietary and physical activity habits [35]. The typical Chinese diet consists of cereals and vegetables with a minimal intake of animal products, limiting calcium availability [50].

A summary of the literature on the beliefs regarding osteoporosis among adolescents and young adults is listed in Table 2.

### 3.3. Practices Affecting Bone Health Among Adolescents and Young Adults

Attaining optimal peak bone mass through healthy diet and lifestyle is critical for osteoporosis prevention [51]. Among the studies, only one showed that subjects actively engaged in good dietary and lifestyle habits to maintain bone health [37]. Chen et al. showed that adolescent females had higher osteoprotective behaviour scores than young adult females in milk drinking, supplement taking and sun exposure [37]. Adolescent males scored higher in avoiding harmful behaviours such as smoking, alcohol, coffee, soft drinks consumption than young adult males [37]. This observation suggests that parental supervision may be effective in enforcing healthy behaviours among adolescents. However, the study identified patterns of behaviour rather than causes, so the predictive value of the variables could not be confirmed.

Seven other studies found that the subjects were not actively engaged in osteoprotective behaviours [15,18,20,30,32,40,44]. Only one study by Barzanji et al. recruited both sexes in their study and they indicated that females were less active physically and less exposed to the sun compared to males [15]. Hence, the investigators urged that there is a need to educate the youngsters about the importance of sunlight in maintaining optimal vitamin D level. It is also important to inform them about the most appropriate time of sun exposure during the day to protect the skin and have the benefit at the same time. Most studies indicated that adolescent females [18,32] or young adults [20,30,40,44] were not engaging in osteoprotective behaviours as evidenced by their dietary or lifestyle habits such as soft drink drinking [18], alcohol or coffee intake [30], lack of calcium intake [18,20,30,40,44] or physical activity [18,20,30,32,44] and less exposure to sun [30].

Besides calcium, vitamin D is another essential nutrient for the development and maintenance of bone. Two studies examined the effects of lifestyle on vitamin D status. Al-Daghri et al. demonstrated a widespread vitamin D deficiency in Saudi Arabian children and adults. They also noticed that frequency of fresh milk consumption was associated with vitamin D levels in the overall population and more specifically in children and female gender [19]. Tonneson et al. showed that the relative risk of vitamin D deficiency was significantly higher (*p* ≤ 0.001) for men (24.9%) compared with women (13.4%) from educational institutions in the Copenhagen area [41]. They also found that obese subjects; subjects who exercised 0–0.5 h a week; and subjects who consumed fast food once a week had higher risk to develop vitamin D deficiency. Smoking was also identified as a risk factor for vitamin D deficiency as chemicals from the cigarette might interfere with vitamin D metabolism [19].

A summary of the literature on the practices affecting bone health among adolescents and young adults is listed in Table 3.

### 3.4. Relationship between Knowledge, Lifestyle and Dietary Habits with Bone Health among Adolescents and Young Adults

Knowledge regarding osteoporosis was crucial in disease prevention. However, only one study reported a significant relationship between knowledge of osteoporosis with bone health indicated by QUS in young women [26]. This suggests more studies need to be done to validate the hypothesis that a higher knowledge level on osteoporosis could translate to better bone health in younger generations.

Physical activity is an important determinant of bone health among the youths [52]. The positive effects of physical activities on various aspects of bone health were illustrated in several studies [27,42]. Weight-bearing physical activity was found to increase both total hip and femoral neck bone mineral density, cortical and periosteal bone volumes in young men [42]. The larger cortical volumes might be contributed by a greater periosteal apposition among the subjects [42]. Bone-specific physical activity (BPAQ) scores was found to be positively correlated with BMD (total hip and femoral neck) of college women but not BMD at L2–L4 [28], suggesting a site-specific effect of those activities. Besides, past physical activity during adolescence was found to be as important as current physical activity for BMD in young adults [29].

Two studies indicated that avoidance of alcohol drinking and cigarette smoking were associated with better bone health [27,42]. In young men, cigarette smoking was associated with lower BMD and QUS indices, but not bone geometry [42]. Meanwhile, moderate alcohol consumption was associated with greater BMD in young men [42]. It is speculated that moderate alcohol intake stimulates the secretion of calcitonin [53] and/or the production of androstenedione by the adrenal which is converted to oestrone [54], thus explaining its bone protective effects. In contrast, Seo et al. found that the higher the alcohol use disorders identification test (AUDIT) scores, the lower the BMD of total femur and femoral neck among young women [27]. However, no significant difference in lumbar spine BMD by alcohol use was found in this study. These findings also supported the idea that the skeletal responsiveness to alcohol may differ by sites [55].

Ensuring sufficient calcium and vitamin D either from food or supplement may also help the younger generations to achieve better bone health [24,26,34,36,46,47,48]. Most studies identified a small but significant positive relationship between calcium intake and BMD or QUS indices [24,46,47,48]. One study found that subjects taking cheese regularly had a significantly higher BMD compared with non-takers [26], highlighting the importance of dairy product besides milk as a source of calcium to maintain bone health. Suriawati et al. showed that individuals with a higher intake of vitamin D alone or in combination with calcium resulted in significantly higher BMC quartiles [34]. A positive correlation between dietary vitamin D and BMD at L2–L4 had also been reported [28]. Some studies reported a negligible association between calcium and vitamin D intake and BMD or the prevalence of osteopenia [29]. This might be caused by under-reporting of the actual calcium and vitamin D intake.

Mu et al. observed a significant decreased risk of osteopenia/osteoporosis among those who practiced calcium food pattern and Chinese traditional pattern among university freshmen [36]. The results suggested that Chinese youths should adhere to a Chinese traditional dietary pattern and increase the intakes of calcium-rich food to maintain good bone health.

A summary of the literature on the relationship between knowledge of osteoporosis, lifestyle and dietary habits with bone health among adolescents and young adults is listed in Table 4.

## 4. Perspectives

The present review showed that the knowledge of adolescents and young adults at school and university depends on formal education, but the general public might rely more on television and internet to learn about osteoporosis. A decrease reliance on health professionals for information on osteoporosis was also noted. Asian adolescents were also dependent on parental guidance to enforce their knowledge and osteoprotective behaviours. Hence, osteoporosis prevention should adopt a multidisciplinary approach to halt the progression of disease starting in youths. It would entail collaboration between medicine, formal education at school and informal education at home. Youths should be taught osteoprotective activities at school, and the parents should be responsible to supervise the osteoprotective habits at home. The switch of preferred health information source, from traditional and reliable sources like the health authority to the modern media calls for the involvement of the authority to engage with the youths via health promotion activities through interactive platform. It also entails health education to empower youths to differentiate between the reliable and unreliable sources of information. Although social media an option to obtain health related information, but there is a need to refer to school education program to get more accurate information.

Besides, most of the adolescents and young adults had low perceived susceptibility and seriousness of osteoporosis, leading to low practice of osteoprotective behaviours. Bone health is influenced by nutrition and lifestyle. Osteoporosis and the associated fractures are preventable by means of adequate nutrition and physical activity [56]. Lifestyle practices are formed early in life and may be carried into adulthood. There is an immediate need to increase osteoporosis awareness and subsequent beliefs, not only in elderly, but also in the younger generation. Awareness creation among the young generations is very important because, in addition to encouraging positive habits to prevent osteoporosis among them, they may also serve as agents to create awareness among their parents and the larger society [4].

Education programs can help in osteoporosis prevention. However, designing education programs for youths requires understanding on their information-seeking behaviours to ensure successful knowledge transfer [57]. One example is through the effective use of traditional and emerging social networks [57]. To address the lack of concern about future disease risk, the health promotion messages could interlace with their current interests in appearance and physical fitness. Successful health promotion should use positive wording to suggest small behavioural changes in diet and food choice that can be incorporated daily. Several education interventions have been implemented to increase the knowledge, beliefs and practices of adolescents or young adults regarding osteoporosis. These approaches include online pre-and post-intervention program [58], circuit training [59] and lecture combine with open discussion [60,61]. The outcome of the intervention was quite promising, however, to what extend that the youths will practice the osteoprotective practice might require further investigation. Besides, education programs regarding the importance of calcium and/or vitamin D have also been carried out [62,63,64]. These interventions utilize slide presentation and interactive group discussion [62], video presentation [63] or class-based nutrition intervention combining traditional lecture and interactive activities [64]. Intervention using slide presentation and interactive group discussion (8 weeks) was noticed to be less effective in changing the dietary calcium and vitamin D intake [62]. However, class-based nutrition intervention combining traditional lecture and interactive activities (15 weeks) successfully increased total milk consumption, specifically fat free milk, and encouraged the switch of milk choice favouring skim milk over low fat milk among students. A summary of the literature on education approaches carried out to improve knowledge, beliefs or practice of adolescents and young adults regarding osteoporosis were listed in Table 5.

There are several limitations regarding this review. Firstly, only two electronic databases were used for literature search, which might limit the number of studies found. Secondly, quality assessment of the studies included in this review is not conducted. In most studies, self-design questionnaires have been used. The reliability and validity of such questionnaires are questionable. The comparability of the studies would be enhanced if all the studies utilized a standard questionnaire for evaluating knowledge, beliefs or practice regarding osteoporosis. However, this was not possible as a myriad of questionnaires had been used. Besides, most of the studies summarized here were local data collected among certain college or university students, hence the results cannot be generalized for the whole nation. Although some of the study participants indicated that they engaged in osteoprotective behaviours, their dietary and supplement intake of nutrients important to bone, duration of physical activities was not explored further. Although some studies showed that participants had poor osteoporosis knowledge, health beliefs and practice on osteoporosis, it was not sure these factors translated to a poorer bone health. Thus, longitudinal studies are needed to examine changes in osteoprotective behaviours and their effects on bone health. A successful intervention program requires the cooperation of public with the researchers. Educational programs are important to deliver general and preventive knowledge of osteoporosis to the public. However, the public need to be motivated to change their diet or health habits to achieve a healthy lifestyle and prevent themselves from getting osteoporosis.

## 5. Conclusions

Adolescents and young adults have poor knowledge regarding osteoporosis. The lack of knowledge regarding osteoporosis leads to low perceived susceptibility and seriousness of osteoporosis. This is because the younger generations think that osteoporosis is a disease affecting only elderly. Perceptions of personal susceptibility and belief in the seriousness of a disease will influence behavioural change in disease prevention. Parental supervision is critical in assuring the practice of osteoprotective behaviours among the adolescents. Healthcare workers may play an important role in planning health education intervention strategies that are suitable for the younger generation to increase their awareness regarding osteoporosis.

## Figures and Tables

**Table 1:** Knowledge regarding osteoporosis among adolescents and young adults.

Studies	Primary Objective	Populations	Locations	Age	Study Design	Findings
**Good knowledge regarding osteoporosis**						
Amre et al. (2008) [31]	To explore baccalaureate nursing students’ knowledge of osteoporosis for beginning practice in the community	85 senior baccalaureate nursing students (58 male & 27 female) in the final 4 years of university	Jordan	19–32 years (mean age 21.74 ± 1.86)	Cross-sectional study using Osteoporosis Knowledge Questionnaire (OKQ) and Osteoporosis Knowledge Test (OKT)	Students had better knowledge regarding prevention of osteoporosis (62.67 ± 14.24) followed by general knowledge of osteoporosis (59.53 ± 20.69) and knowledge regarding pathophysiology of osteoporosis (39.66 ± 13.65)
Chen et al. (2012) [37]	To examine demographic characteristics, knowledge, and attitudes of adolescents on osteoprotective practices in Taichung City, Taiwan	329 (120 male and 209 female) randomly selected high schools and colleges students	Taichung City, Taiwan	Adolescents (senior high school students) and young adults (undergraduates)	Cross-sectional study using Osteoporosis Knowledge Scale (OKS)	Adolescent males and females obtained a higher score than their adult counterparts.
Khan et al. (2014) [33]	To evaluate knowledge and perceptions of osteoporosis among university students in Malaysia	461 students (214 male and 247 female): 165 Malay, 147 Chinese, 125 Indian, 24 Others	Universiti Sains Malaysia (USM) Penang, Malaysia	Mean age 24.61 ± 5.51 years	Cross-sectional study using a pre-validated self-design questionnaire was used to assess the knowledge regarding osteoporosis	87% identified osteoporosis correctly as a disease that makes bones weak and fragile Chinese scored the highest in knowledge section followed by the Malays and Indians.
Uddin et al. (2013) [39]	To understand the level of gap of knowledge and awareness regarding the essentiality of calcium and vitamin D among pharmacy students at undergraduate level in Bangladesh	713 (350 male and 363 female) undergraduate pharmacy students from different public and private universities	Dhaka and Chittagong cities of Bangladesh, India	Age 18 to 20 years	A questionnaire was devised to get a gross idea about the preliminary and basic knowledge on calcium and vitamin D	82.2% students know about the term osteoporosis Male and female students equally knew the importance of calcium (98.6% vs. 99.2%) and vitamin D (99.4% vs. 99.2%) for bone health
**Poor knowledge regarding osteoporosis**						
Bilal et al. (2017) [44]	To assess knowledge, attitudes and practices about osteoporosis among female medical school entrants in Karachi	400 female medical school entrants of DOW University of Health Sciences (DUHS) and Jinnah Sindh Medical University (JSMU)	Karachi, Pakistan	Mean age 19.4 ± 1.2 years	Cross-sectional study using a pre-validated Osteoporosis Knowledge Assessment Tool (OKAT)	Only 8.0% of the participants had a good score pertaining to knowledge about osteoporosis whereas majority of the participants (49.0%) had a poor score
Ediriweera de Silva et al. (2014) [20]	To determine the knowledge, beliefs and practices regarding osteoporosis among young females entering medical schools in Sri Lanka	186 female medical school entrants	Faculties of Medicine, Universities of Colombo and Kelaniya, Sri Lanka	Mean age 20.7 ± 2.1 years	Cross-sectional study using a validated Osteoporosis Knowledge Assessment Tool (OKAT)	Majority of the young adults (51.6%, *n* = 96) had an average score (40/ 60) on the knowledge test, while 40.8% (*n* = 76) had a poor score (<40) The knowledge of osteoporosis risk factors and preventive practices among participants were shown to be poor
Gammage et al. (2011) [21]	To examine gender differences in osteoporosis-related knowledge in university students	527 participants (351 women & 176 men) from first-year university courses in a kinesiology department	Brock University, St. Catharines, Ontorio, Canada	College-aged	Cross-sectional study using Osteoporosis Knowledge Tool (OKT)	Knowledge regarding osteoporosis was generally poor When compared among gender, women showed significantly higher (*p* ≤ 0.001) total general knowledge scores regarding osteoporosis (61.35 ± 1.77) than men (57.67 ± 12.40)
Ghaffari et al. (2015) [43]	To investigate the health faculty students’ awareness of osteoporosis (calcium intake and physical activity).	228 female undergraduate students in the health faculty, Shahid Beheshti University of Medical Sciences	Iran	18–24 years old (mean age 22.17 ± 2.66 years)	A validated questionnaire to assess knowledge regarding osteoporosis was used	Only 19.2% of the students had a high-level knowledge of osteoporosis Mean score of knowledge regarding osteoporosis was 12.96 ± 4.01 (4.67 ± 1.66 for calcium intake and 8.29 ± 2.89 for physical activity section)
Njeze et al. (2017) [4]	To determine the awareness of osteoporosis and factors that determine awareness of osteoporosis	500 respondents from a polytechnic	Enugu, South East Nigeria	Less than 20 to 51 years old and above (mean age 26.5 ± 7.4 years)	Cross-sectional study using a self-design questionnaire	Only 6.3% of the adolescents (<20 years old) answered the questions about knowledge of osteoporosis correctly 4.4% of the young adults (21–30 years old) answered the questions about knowledge of osteoporosis correctly
Sayed-Hasaan, Bashour and Koudsi (2013) [38]	To determine the level of osteoporosis knowledge and beliefs among nursing college students in Damascus	353 female students in nursing college	Damascus, Syria	Mean age 19.9 years	Cross-sectional study using osteoporosis-related tools (Arabic version), namely the Osteoporosis Knowledge Assessment Tool (OKAT)	Very poor knowledge among the young adults about risk factors of osteoporosis, such as post-menopausal status as a period of accelerated bone loss, family history of osteoporosis and related fractures. Young adult females achieved total mean knowledge score of 7.9 (2.7) out of possible 20 points, being 39.6% of possible maximum score on the OKAT
**Difference in osteoporosis knowledge based on sex, study program and country**						
Alamri et al. (2015) [17]	To assess knowledge, attitude, and practices for osteoporosis among Saudi general population and to identify its determining factors	1830 respondents (1062 male and 728 female)	Every region in the Kingdom of Saudi Arabia	Age 18 years or older (Mean age 37.1 ± 14.3 years in male & 36.3 ± 13.6 years in female)	Cross-sectional study using a pre-validated self-design questionnaire was used to assess the knowledge regarding osteoporosis	78% of the respondents heard about osteoporosis Female respondents were more knowledgeable compared to male The main sources of knowledge were healthcare providers (27%), followed by family member or friend (23.7%), then through internet (21.5%) and TV (19%). This was consistent for both men and women, except for TV as a source of knowledge, which came in the second place for only women
Ford et al. (2011) [35]	To differences in osteoporosis knowledge, self-efficacy, and health beliefs among China and American college students	408 US and 409 Chinese undergraduate students (342 male, 468 female) from University of Mississippi Withheld During Review, United States and Tianjin Medical University Withheld During Review, Peoples Republic of China respectively	United States and China	Mean age 19.38 ± 1.25 years	Validated Osteoporosis Knowledge Test (OKT) was used	US students had significantly higher (*p <* 0.05) total knowledge (14.52 ± 4.16 vs. 11.82 ± 3.76), exercise knowledge (9.04 ± 3.17 vs. 7.27 ± 2.67) and calcium knowledge (9.73 ± 3.35 vs. 7.23 ± 2.71) than Chinese students The scores for exercise knowledge and calcium knowledge subscales were low, as were the total osteoporosis knowledge scores: Total knowledge score: US 60.5%, Chinese 49.2% correct Exercise knowledge score: US 56.5%, Chinese 45.4% Calcium knowledge score: US 57.2%, 42.5%
Nguyen and Wang (2012) [45]	To investigate osteoporosis knowledge in students who were soon to be nurses, pharmacists, physical therapists, and dietitians	206 female students of University of Missouri	Columbia	21 to 27 years	Revised version of Osteoporosis Knowledge Test (OKT) was used	On the total OKT scale, 2nd year pharmacy students correctly answered about 19.48 items out of the 32 items, which was significantly lower than all other students except 1st year physical therapy students Senior dietetics students correctly answered 24.40 items on the total OKT scale, the highest performing group of the eight classes Comparing the two classes within the same program, the two classes in pharmacy (2nd and 4th year) were statistically significantly different, as were the two classes in physical therapy (1st and 3rd year). There were discrepancies in specific areas of osteoporosis knowledge between the classes of students, and the average scores of correctly answered items were only as high as 24.40 (76.3%) out of 32 items on osteoporosis knowledge.
**Sources of osteoporosis knowledge**						
Al-Zu’bi, Almuhtaseb and Amayreyh (2010) [32]	To assess the knowledge in a group of teenage girls about risk factors and lifestyle affecting osteoporosis	320 girls attending the 8–10th grade	Schools from east and west of Amman, the capital city in Jordan	13–17 years (mean age 14.4 ± 0.9 years)	Self-design questionnaire to assess knowledge of risk factors regarding osteoporosis was used	84.3% of the young girls have heard about osteoporosis. Media especially the television (46.2%) was a primary source of information among teenagers. Family (mothers and grandmothers) role was evident in 30% of the cases. The role of the schools was minimal in only about 16% of the cases. Reading was the least popular option of scientific information (only 7%)
Barzanji, Alamri and Mohamed (2013) [15]	To assess the awareness of adults in Riyadh about osteoporosis and its associated factors as well as compare knowledge, attitude and practice levels of men and women	505 participants from eight malls	Riyadh city, Saudi Arabia	Mean age 33.78 ± 10.46 years	Cross-sectional study using pre-coded Arabic questionnaire	The mean knowledge score of osteoporosis among young adults was 13.55 ± 3.996 (*n* = 310) out of a total possible maximum score of 24 The knowledge score was significantly associated with education, employment, income and residence (*p <* 0.05) Sources of knowledge about osteoporosis were from television (56%), followed by relatives and friends (25%), newspapers (24%) and the least was from health care providers (18%)
Puttapitakpong et al. (2014) [25]	To assess the inter-correlation of knowledge, attitude and osteoporosis preventive behaviours in women around the age of peak bone mass.	430 women attending the Gynecology Clinic	King Chulalongkorn Memorial Hospital, Bangkok, Thailand	20–35 years’ old	Cross-sectional study using a pre-validated self-design questionnaire	Only 49.5% of the participants had heard about osteoporosis. Most of them learnt it from television (95.3%, *n* = 203/213) and the internet (72.8%, *n* = 155/213) Only 30% of them obtained the information from a doctor, nurse or midwife

**Table 2:** Beliefs regarding osteoporosis among adolescents and young adults.

Studies	Primary Objective	Populations	Location	Age	Study Design	Findings
**Perceived high susceptibility regarding osteoporosis (men > women)**						
Alamri et al. (2015) [17]	To assess knowledge, attitude, and practices for osteoporosis among Saudi general population and to identify its determining factors	1830 respondents (1062 male and 728 female)	Every region in the Kingdom of Saudi Arabia	Age 18 years or older (Mean age 37.1 ± 14.3 years in male & 36.3 ± 13.6 years in female)	Cross-sectional study using a pre-validated self-design questionnaire was used to assess the health belief regarding osteoporosis	Perceived susceptibility (men: 3.9 ± 1.4; women: 3.5 ± 1.3) and benefits (men: 2.8 ± 1.2; women: 2.6 ± 1.1) regarding osteoporosis were higher among men than women No statistically significant differences in the mean levels of perceived severity (3.9 ± 1.2) and barriers (6.7 ± 1.5) regarding osteoporosis between men and women
**Perceived high susceptibility regarding osteoporosis (women > men)**						
Ford et al. (2011) [35]	To differences in osteoporosis knowledge, self-efficacy, and health beliefs among China and American college students	408 US and 409 Chinese undergraduate students (342 male, 468 female) from University of Mississippi Withheld During Review, United States and Tianjin Medical University Withheld During Review, Peoples Republic of China respectively	United States and China	Mean age 19.38 ± 1.25 years	Validated Osteoporosis Health Belief Scale (OHBS) was used	Female students had a greater perception of susceptibility than their male counterparts (female: 13.88 ± 4.53, male: 12.24 ± 4.41) Barriers to exercise and calcium intake were greater for the Chinese students.
Gammage et al. (2011) [21]	To examine gender differences in osteoporosis-related knowledge in university students	527 participants (351 women & 176 men) from first-year university courses in a kinesiology department	Brock University, St. Catharines, Ontorio, Canada	College-aged	Cross-sectional study using Osteoporosis Health Belief Scale (OHBS)	Women reported significant higher (*p* ≤ 0.001) osteoporosis-related beliefs: susceptibility (2.42 ± 0.91), seriousness (3.35 ± 0.84), calcium barriers (2.14 ± 0.86) as compared to men’s beliefs regarding osteoporosis: susceptibility (1.69 ± 0.73), seriousness (3.07 ± 0.93), calcium barriers (1.76 ± 0.71) Women reported significant lower (*p* ≤ 0.001) exercise self-efficacy (77.82 ± 15.08) as compared to men (84.51 ± 13.90)
Shanthi et al. (2008) [22]	To compare osteoporosis health beliefs among different age and gender groups	300 participants (45 male and 97 female: 18–25 years old)	University town in Canada	Three age groups (18 to 25, 30 to 50, and 50-plus)	Cross-sectional study using Osteoporosis Health Belief Scale (OHBS) questionnaire	Women scored higher in perceived susceptibility towards osteoporosis than men in the age group of 18–25 years old (10.86 ± 4.01 vs. 8.58 ± 3.38) No significant differences in perceived seriousness and health motivation scores across age and gender groups
**Perceived low susceptibility regarding osteoporosis**						
Bilal et al. (2017) [44]	To assess knowledge, attitudes and practices about osteoporosis among female medical school entrants in Karachi	400 female medical school entrants of DOW University of Health Sciences (DUHS) and Jinnah Sindh Medical University (JSMU)	Karachi, Pakistan	Mean age 19.4 ± 1.2 years	Cross-sectional study using a pre-validated Osteoporosis Health Belief Scale (OHBS)	Perceived susceptibility was low as only 14.0% of the participants believed that they were at a high risk for osteoporosis More than half of the participants believed that osteoporosis is a serious disease, while more than three quarters of them considered it to be a barrier in their daily routines
Chen et al. (2012) [37]	To examine demographic characteristics, knowledge, and attitudes of adolescents on osteoprotective practices in Taichung City, Taiwan	329 (120 male and 209 female) randomly selected high schools and colleges students	Taichung City, Taiwan	Adolescents (senior high school students) and young adults (undergraduates)	Cross-sectional study using Osteoporosis Attitude Scale (OAS) questionnaire	Adolescent males had a lower score compared to young adult males on the subscale of suffering from osteoporosis (7.88 ± 2.52 vs. 9.17 ± 2.23) but a higher score on the subscale of prevention (17.07 ± 2.35 vs. 15.76 ± 2.14), the similar trends were also observed between adolescent females and young adult females (suffering: 9.25 ± 2.28 vs. 9.44 ± 2.39, prevention: 16.81 ± 2.09 vs. 16.70 ± 2.71)
Ediriweera de Silva et al. (2014) [20]	To determine the knowledge, beliefs and practices regarding osteoporosis among young females entering medical schools in Sri Lanka	186 female medical school entrants	Faculties of Medicine, Universities of Colombo and Kelaniya, Sri Lanka	Mean age 20.7 ± 2.1 years	Cross-sectional study using Osteoporosis Health Belief Scale (OHBS) questionnaire	Only 13.9% (*n* = 26) of women agreed that their chances of getting osteoporosis are high About 53.7% (*n* = 100) of the participants felt that if they had osteoporosis, it would change their whole life 54.8% (*n* = 102) of the participants mentioned that the thought about osteoporosis scared them 83.3% (n = 155) of them felt that having osteoporosis would make their life difficult
Khan et al. (2014) [33]	To evaluate knowledge and perceptions of osteoporosis among university students in Malaysia	461 students (214 male and 247 female): 165 Malay, 147 Chinese, 125 Indian, 24 Others	University Sains Malaysia (USM) Penang, Malaysia	Mean age 24.61 ± 5.51 years	Cross-sectional study using a pre-validated self-design questionnaire was used to assess the perceptions regarding osteoporosis	Study participants had a low perception on seriousness of osteoporosis compared to cancer and diabetes
Sayed-Hasaan, Bashour and Koudsi (2013) [38]	To determine the level of osteoporosis knowledge and beliefs among nursing college students in Damascus	353 female students in nursing college	Damascus, Syria	Mean age 19.9 years	Cross-sectional study using Osteoporosis Health Belief Scale (OHBS) questionnaire	Students believed osteoporosis is a serious disease but did not feel susceptible to or concerned about the illness. Despite having a positive view regarding calcium intake and physical activity, young women in the study perceived moderate to high barriers to exercises and calcium intake.
**Barrier to osteoprotective behaviours**						
Marcinow et al. (2017) [23]	To determine young adults’ knowledge of calcium in relation to health, facilitators and barriers to adequate calcium intake	53 participants (18 male and 35 female)	Communities in Guelph and surrounding areas, Ontorio, Canada	18–34 years’ old	Attitudes and Beliefs Focus Group Study, by using a semi-structured interview guide, guided by social cognitive theory	Perceived barriers to calcium intake included high cost, inconvenience of milk products and negative practices of dairy farmers.

**Table 3:** Practices affecting bone health among adolescents and young adults.

Studies	Primary Objective	Populations	Location	Age	Study Design	Findings
**Actively engaged in osteoprotective behaviours**						
Chen et al. (2012) [37]	To examine demographic characteristics, knowledge, and attitudes of adolescents on osteoprotective practices in Taichung City, Taiwan	329 (120 male and 209 female) randomly selected high schools and colleges students	Taichung City, Taiwan	Adolescents (senior high school students) and young adults (undergraduates)	Cross-sectional study using Osteoporosis Lifestyle Scale (OLS) questionnaires	Adolescent females had higher osteoprotective behaviour scores than young adult females in milk drinking (3.28 ± 1.00 vs. 2.96 ± 1.02), supplement taking (2.05 ± 0.96 vs. 1.76 ± 0.95), and sun exposure (3.64 ± 1.06 vs. 3.20 ± 1.03). Adolescent males scored higher in avoiding harmful behaviours such as smoking, alcohol, coffee, soft drinks consumption than did young adult males (18.65 ± 2.31 vs. 17.13 ± 3.32)
**Not actively engaged in osteoprotective behaviours**						
Al-Raddadi et al. (2018) [18]	To estimate the prevalence of behaviours affecting bone health and vitamin D status and to identify factors associated with vitamin D deficiency among Saudi adolescent females	421 female adolescents	Secondary schools in Jeddah City, Saudi Arabia	Mean age 17.2 ± 1.2 years	Cross-sectional study using self-design questionnaire	Almost half of the participants (46.1%) reported very low consumption of dairy products 62.9% of the participants reported drinking canned soft drinks 1–4 cans/week Only 12.4% of the participants were taking calcium and vitamin D supplements. 49.4% of them reported avoidance of sun exposure Almost half (43.7%) of the participants did not perform any exercise, and only 13.9% performed the recommended exercise per week
Al-Zu’bi, Almuhtaseb & Amayreyh (2010) [32]	To assess the knowledge in a group of teenage girls about risk factors and lifestyle affecting osteoporosis	320 girls attending the 8–10th grade	School from east and west of Amman, the capital city in Jordan, Arab	13–17 years (mean age 14.4 ± 0.9 years)	Self-design questionnaire to assess lifestyle of students	62.8% of the students reported eating dairy products frequently Around 68% of them do not participate in any regular exercise. 43.4% of them avoid exposure to the sun.
Barzanji, Alamri and Mohamed (2013) [15]	To assess the awareness of adults in Riyadh about osteoporosis and its associated factors as well as compare knowledge, attitude and practice levels of men and women	505 participants from eight malls	Riyadh city, Saudi Arabia	Mean age 33.78 ± 10.46 years	Cross-sectional study using pre-coded Arabic questionnaire	Only 10% of females have adequate physical exercise in comparison to 23% of males 22% of females had no exposure to sun, in comparison to 3% of males
Bilal et al. (2017) [44]	To assess knowledge, attitudes and practices about osteoporosis among female medical school entrants in Karachi	400 female medical school entrants of DOW University of Health Sciences (DUHS) and Jinnah Sindh Medical University (JSMU)	Karachi, Pakistan	Mean age 19.4 ± 1.2 years	Cross-sectional study using self-design questionnaire	The RDA for calcium was equal to or greater than 700 mg per day which was met by only 29.0% of the participants despite of the high motivation towards consuming a calcium rich diet Exercise levels were insufficient in terms of both, duration and the recommended type of exercise. Only 12.0% of the participants engaged in exercises based on the recommended guidelines. Only 5.5% subjects were involved in definitive behaviours to improve bone health
Ediriweera de Silva et al. (2014) [20]	To determine the knowledge, beliefs and practices regarding osteoporosis among young females entering medical schools in Sri Lanka	186 female medical school entrants	Faculties of Medicine, Universities of Colombo and Kelaniya, Sri Lanka	Mean age 20.7 ± 2.1 years	Cross-sectional study using modified validated food frequency questionnaire and questions regarding positive and negative behaviours towards osteoporosis	Only 35 (18.8%) of the participants achieved the Recommended Daily Allowance for calcium. Only 23 (13.6%) of the participants engaged in the recommended exercises in type and duration
Park et al. (2015) [30]	To examine dietary intakes and patterns, health behaviours in relation to obesity and bone mineral density (BMD)	160 females nursing students	College in Seoul, Republic of Korea	Mean age 20.6 ± 1.48	Cross-sectional study examines dietary habits (3- day food dairy collection) and health behaviours	90% (*n* = 144) of them reported as alcohol and coffee consumers 76% (*n* = 122) students had low milk drinking 63.1% of the students admitted that they were not engaged in regular exercise
Sidor, Glabska & Wlodarek (2016) [40]	To analyze the osteoporosis risk, based on diet assessment in young Polish women	75 young Polish women	Warsaw, Poland	20–30 years (mean age 24.1 ± 3.4)	Three-day dietary record was used	Only 25% had an adequate intake of calcium and, while supplementation was considered, 10% had an adequate intake of vitamin D.
**Effect of lifestyle on vitamin D status**						
Al-daghri et al. (2015) [19]	To investigate vitamin D status and its association with consumption frequencies of various dairy products in Saudi population	820 adolescents and 565 young adults	Different primary health care centers within Riyadh, Saudi Arabia	Adolescents: 327 boys (mean age 14.9 ±1.6 years) and 493 girls (mean age 14.8 ± 1.6) Young Adults: 249 men (mean age 27.9 ± 0.8) and 316 women (32.2 ± 0.6)	A qualitative food frequency questionnaire was used	Adolescents: 80% of boys and 90% of girls had deficient/insufficient levels of vitamin D Young adults: 64% of men and 50% of women had deficient/insufficient levels of vitamin D Frequency of overall dairy product consumption was significant only in women (*p* < 0.05) and this association was lost after adjusting for age and BMI Frequency of fresh milk consumption affected vitamin D levels in the overall population and more specifically in children and female gender (*p* ≤ 0.001)
Tonneson et al. (2016) [41]	To investigate the association between lifestyle and vitamin D status in a sample of untreated young adults	738 young adults (361 male and 339 female)	Educational institutions in the Copenhagen area, Denmark	Women: mean age 22 ± 2.2 years Men: mean age 21.6 ± 2.3 years	Cross-sectional study assessing exercise and smoking habits, alcohol intake and dietary habits	The relative risk (RR) for vitamin D deficiency was highest for men 2.09 (1.52, 2.87); obese subjects 2.00 (1.27, 3.15); smokers 1.33 (1.02, 1.73); subjects who exercised 0–½ h a week 1.88 (1.21, 2.94); and subjects who consumed fast food once a week 1.59 (1.05, 2.43)

**Table 4:** Relationship between knowledge of osteoporosis, lifestyle and dietary habits with bone health among adolescents and young adults.

Studies	Primary Objective	Populations	Location	Age	Study Design	Findings
**Effect of knowledge regarding osteoporosis on bone health**						
Iwasaki et al. (2013) [26]	To investigate the influence of lifestyle on bone mineral density (BMD) and bone turnover among young women in Chiang Mai, Thailand	177 healthy women	Chiang Mai University hospital, Chiang Mai, Thailand	20–30 years (mean age 23.4 ± 2.5)	Modified version of the Osteoporosis Knowledge Test (OKT) was used	Normal group (Higher BMD) had a tendency of better osteoporosis knowledge regarding calcium (4.9 ± 1.6) and vitamin D (1.7 ± 0.7) compared to Low BMD 1 (calcium: 4.1 ± 1.6; vitamin D: 1.5 ± 0.8) and Low BMD 2 (calcium: 4.8 ± 2.1; vitamin D: 1.6 ± 0.7) groups.
**Effect of lifestyle habits (physical activity, smoking, alcohol drinking) on bone health**						
Eleftheriou et al. (2013) [42]	The association of smoking, alcohol consumption and prior exercise with lower limb bone volume, composition and structure in a large cohort of healthy Caucasian males	723 healthy male military recruits on entry to Army training	United Kingdom	16–18 years (mean age 19.92 ± 0.09 years)	Self-design questionnaire used to assess lifestyle factors	Weight-bearing physical activity enhanced periosteal bone apposition, increases in both total hip and femoral neck bone mineral density (BMD; *p* ≤ 0.001 in both cases), and cortical (*p* = 0.016) and periosteal bone volumes (*p* ≤ 0.001) Smoking was detrimental to bone mineral density and QUS measures, but not proximal femoral geometry Moderate alcohol consumption was associated with greater BMD (*p* ≤ 0.001)
Seo et al. (2015) [27]	To assess the association between alcohol consumption and healthy Korean young women bone	1176 healthy women	Sahmyook University, Seoul, South Korea	19–30 years (mean age 24.68 ± 0.12 years)	Cross-sectional study by Alcohol Use Disorders Identification Test (AUDIT) scores and drinking consumption; frequency and amount	The BMD of total femur (TF) and femoral neck (FN) was lower with higher alcohol use disorders identification test (AUDIT) scores Those who drink more frequently were more likely to have lower BMD at femoral neck (FN). This difference in FN BMD became more significant between abstainers and young women who were weekly and monthly drinkers and drank more than five glasses per occasion
**Effect of past physical activity on bone health**						
Kim et al. (2016) [28]	To investigate the relationship between bone-specific physical activity (BPAQ) scores, body composition, and bone mineral density (BMD) in healthy young college women	73 college women	Universities in Seoul and Gyeonggi province, South Korea	19–26 years (mean age 21.7 ± 1.8 years)	Cross-sectional study using food intake questionnaire and bone-specific physical activity questionnaire	Bone-specific physical activity (BPAQ) scores was positively correlated with bone mineral density (total hip and femoral neck) but no correlation with L2–L4 There was a positive correlation between dietary Vitamin D and L2–L4 (*p* = 0.025)
Kim et al. (2013) [29]	To determine factors associated with the bone mineral density (BMD) of university students	111 male students from School of Medicine	Chung-Ang University, College, Seoul, South Korea	19–34 years (Mean age 23.2 years	Global Physical Activity Questionnaire and food frequency questionnaire (FFQ) were used	Past physical activity during adolescence (*p* = 0.002) showed a positive effect on the bone mineral content In the multivariate model, past physical activity (≥1 time/week) had a protective effect on osteopenia (prevalence ratio, 0.37; 95% confidence interval (CI), 0.18 to 0.75) and present physical activity (1000 metabolic equivalent of task-min/week) decreased the risk of osteopenia (prevalence ratio, 0.64; 95% CI, 0.44 to 0.91) Calcium and vitamin D intake did not affect the BMD or the prevalence of osteopenia
**Effect of dietary habit (calcium intake) on bone health**						
Chouinard, Simpson and Buchholz (2012) [24]	To identify predictors of bone mineral density (BMD) in young, healthy adults	261 (77 male and 184 female) subjects	University of Guelph in southwestern Ontario, Canada	18–33 years (mean age 21.4 ± 2.1 years)	Cross-sectional study using physical activity questionnaire for adults (PAQ-AD) and self-administered food frequency questionnaire (FFQ)	Men: BMD at the total hip, femoral neck, and total body was positively predicted by body mass, weekly frequency of participation in weight-bearing physical activities, and calcium intake Weekly frequency of weight-bearing physical activities positively predicted spine BMD. Women: Body mass alone positively predicted log BMD of the total body and spine Body mass and calcium intake positively predicted log BMD of the femoral neck. Log BMD of the total hip was positively predicted by body mass and the absence of a family history of osteoporosis
Hervas et al. (2018) [46]	To analyze the relationship of physical activity (PA), physical fitness, body composition, and dietary intake with bone stiffness index (SI) in young university students	156 (61 male, 95 female) young adults from different university degree programs	University of the Basque Country	18–21 years old (mean age 18.74 ± 0.77 years)	Five days’ dietary record was used	Males’ calcium intake (1018 mg/day ± 348) reached the recommended value, the females’ average (814 mg/day ± 206) Neither males (3.88 mg/day ± 2.38) nor females (3.10 mg/day ± 2.23) reached an adequate vitamin D intake (5 mg/day). Calcium consumption (*p* < 0.001) and vitamin D intake (*p* = 0.021) were higher in males In the overall group, calcium intake showed a positive correlation with SI (*p* = 0.022)
Ito et al. (2011) [47]	To examine habitual phosphorus and calcium intake and the calcium/phosphorus intake ratio on the bone mineral density (BMD) in young Japanese women	441 first-year female students of Kagawa Nutrition University	Japan	18–22 years	Dietary habits during the preceding month were assessed using diet history questionnaire (DHQ)	Calcium intake and the calcium/phosphorus intake ratio independently had positive and significant associations with BMD in the distal radius adjusted for postmenarcheal age, body mass index, and physical activity No significant associations of calcium intake and the calcium/phosphorus intake ratio independently with BMD in the lumbar spine and femoral neck
Iwasaki et al. (2013) [26]	To investigate the influence of lifestyle on bone mineral density (BMD) and bone turnover among young women in Chiang Mai, Thailand	177 healthy women	Chiang Mai University hospital, Chiang Mai, Thailand	20–30 years (mean age 23.4 ± 2.5)	Self-design questionnaire consists of lifestyle-relating factors: eating habits, diet history and exercise experience	Subjects in the regular cheese intake “yes” group (110 ± 23.3) had a significantly (*p* < 0.05) higher BMD compared with the “no” group (99.7 ± 17)
Liberato, Bressan and Hills (2013) [48]	To examine the relationship between dietary factors and physical activity on bone mineralization in young men.	35 men from the local community	City of Brisbane, Australia	18–25 years	Cross-sectional study where food intake was assessed using household estimates in a food record	Higher BMC was observed in whole body, trunk and lumbar regions but not in legs or arms of young men who consumed more than 1000 mg/day of calcium compared to those who consumed less than 1000 mg/day of calcium
Mu et al. (2014) [36]	To examine associations between dietary patterns and body mass index (BMI) and bone mineral density (BMD) in Chinese freshmen.	1319 college freshmen	4 universities in Hefei, China	16–20 years (mean age 18.1 ± 1.2 years)	Cross-sectional study using modified food-frequency questionnaire	The calcium food pattern and Chinese traditional pattern were associated with a decreased risk of osteopenia/osteoporosis before and after adjusting for confounders (*p* < 0.05)
Suriawati et al. (2016) [34]	To investigate the relationship between the dietary intake of calcium and vitamin D, physical activity, and bone mineral content (BMC) in 13-year-old Malaysian adolescents	289 adolescents (99 male, 190 female) school children from selected public secondary schools from the central and northern regions of Peninsular Malaysia)	Malaysia	13-year-old	Cross-sectional study using seven-day diet histories questionnaire	The average dietary intakes of calcium and vitamin D were 377 ± 12 mg/day and 2.51 ± 0.12 µg/day, respectively, with most subjects failing to meet the Recommended Nutrient Intake (RNI) of Malaysia for dietary calcium and vitamin D Subjects with a higher intake of vitamin D, a higher combination of the intake of vitamin D and calcium had significantly higher BMC quartiles

**Table 5:** Education approaches to improve knowledge, beliefs or practice of adolescents and young adults regarding osteoporosis.

Studies	Design	Location	Setting	Population	Intervention Descriptions	Outcomes
**Educational intervention to modify knowledge, attitude and practices towards osteoporosis**						
Schoenfeld et al. (2010) [58]	Tailored Web-Education System (TWEEDS) Tool and Web Site Development	New York	School	*n* = 89, age 13 to 17 years, mean age 15.7 years	Online pre- and postintervention surveys (45 min) evaluated participants’ pre- and postintervention osteoporosis knowledge, attitudes, preventive practices, and postintervention intent to change healthy bone practices. Participants completed the Web-based program that provided detailed information about osteoporosis, and healthy bone practices, immediately after completing the pre-test and just prior to completing the post-test	Adolescents changed their perception regarding the disease seriousness and considered adopting osteoporosis prevention practices
Sanaeinasab et al. (2013) [59]	Quasi-experimental study	Female students resided in a town near Tehran	School	*n* = 45, 15 to 16 years’ old	Three group sessions of 60 min per week educational programme based on the Health Belief Model. Lecture, question and answer, brain-storming, group discussion with pamphlets about the role of nutrition and physical activity in disease prevention and a booklet on osteoporosis.	Before intervention, only 2.2% of the subjects have good knowledge regarding osteoporosis, it increases to 66.7% after intervention Mean scores of some Health Belief Model structures (perceived susceptibility towards osteoporosis, perceived barriers of physical activity, self-efficacy of calcium and physical activity) changed significantly after the intervention (*p* < 0.05) Post-intervention, physical activity increased (*p* = 0.041) but calcium intake did not.
Takahata (2018) [60]	Circuit exercise training	Baika Women’s University, Osaka, Japan	School	*n* = 41, mean age 18.5 ± 0.6 years	Circuit training which involves performing both anaerobic and aerobic exercise continuously for 3 months (5 mins × 3 sets =15 mins, do the exercise at least 3 days in a week)	Broadband ultrasound attenuation of the calcaneus was higher 2 months later (*p* = 0.033) as well as 3 months later (*p* = 0.036), and the speed of sound of the calcaneus was higher 3 months later (*p* = 0.018) in the exercising group Muscle mass was strongly positively correlated with the calcaneus QUS-SOS.
Zhang et al. (2012) [61]	One group quasi-experimental study	Shaanxi, Northwest China	Nursing school	*n* = 256, mean age 18.80 ± 1.55 years	2.5-h lecture followed by 30 min open discussion, and 20 min for questions and answers. The lecturer summarized the content matter delivered during the program following the question-and-answer period to reinforce teaching and learning objectives. The educational program addressed the definition, prevalence, and etiology of osteoporosis; risk factor identification; physical signs of the disease; preventive and diagnostic measures; and treatment	Intervention successfully increased the baseline osteoporosis knowledge score two weeks after the educational in-service The educational program significantly increased total osteoporosis health beliefs and the subscales, except for the perceived barriers to exercise and calcium intake
**Educational intervention to improve Calcium and/or Vitamin D intake**						
Bohaty et al. (2008) [62]	Convenience sampling method	Lincoln, Nebraska, and Ankeny, Iowa.	Day-care center	*n* = 80, 19 to 29 years, mean age 22.3 ± 3.1 years	8 weeks, ten 45-min slide show presentations on the importance of dietary intake of calcium and vitamin D in preventing osteoporosis. The slide show was followed by an interactive group discussion regarding problems with increasing dietary intake of calcium and vitamin D. After the intervention, subjects received a packet to take home that included an educational handout from the NOF (n.d.) and an outline of the slide show presentation.	Post-test scores on knowledge of osteoporosis, calcium, and vitamin D were significantly higher than pre-test scores 8 weeks after the educational intervention There was no change in dietary intake of calcium, vitamin D, and dairy products from pre- to post-intervention
Goodman, Morrongiello & Meckling (2016) [63]	Randomized controlled trial	Guelph and throughout Ontario.	Community	*n* = 90, 18 to 25 years	The intervention group watched a video, received online information and tracked intake of vitamin D using a mobile application for 12 weeks.	The increase in vitamin D knowledge from time 1–3 was significantly higher in the intervention than control group (t (88) = 2.26, *p* = 0.03) The intervention group (M = 3.52, SE = 0.13) had higher overall perceived importance of vitamin D supplementation than the control (M = 3.16, SE = 0.12), F (1, 88) = 4.38, *p* = 0.04, *ηp* 2 = 0.05.
Ha et al. (2009) [64]	Class based nutrition intervention	Midwest university	School	*n* = 80, 18 to 24 years	15 weeks’ class lectures (3 times per weeks for 50 min) focused on healthful dietary choices related to prevention of chronic diseases and were combined with interactive hands on activities and dietary feedback	Class-based nutrition intervention combining traditional lecture and interactive activities was successful in decreasing soft drink consumption Total milk consumption, specifically fat free milk, increased in females and male students changed milk choice favouring skim milk over low fat milk. (1% and 2%)

## References

[1] Yamamoto K. (2001). Definition and diagnostic criteria of osteoporosis in Japan. Clin. Calcium.

[2] Svedbom A., Ivergård M., Hernlund E., Rizzoli R., Kanis J.A. (2014). Epidemiology and economic burden of osteoporosis in Switzerland. Arch. Osteoporos..

[3] Wade S.W., Strader C., Fitzpatrick L.A., Anthony M.S., O’Malley C.D. (2014). Estimating prevalence of osteoporosis: Examples from industrialized countries. Arch. Osteoporos..

[4] Njeze Ngozi R., Ikechukwu O., Miriam A., Olanike A.-U., Akpagbula Ulugo D., Njeze Nneze C. (2017). Awareness of Osteoporosis in a Polytechnic in Enugu, South East Nigeria. Arch. Osteoporos..

[5] NIH Consensus Development Panel (2001). Osteoporosis Prevention, Diagnosis, and Therapy. JAMA.

[6] Stagi S., Cavalli L., Iurato C., Seminara S., Brandi M.L., de Martino M. (2013). Bone metabolism in children and adolescents: Main characteristics of the determinants of peak bone mass. Clin. Cases Miner. Bone Metab..

[7] O’Brien M. (2001). Exercise and osteoporosis. Irish J. Med. Sci..

[8] McKay H.A., Bailey D.A., Mirwald R.L., Davison K.S., Faulkner R.A. (1998). Peak bone mineral accrual and age at menarche in adolescent girls: A 6-year longitudinal study. J. Pediatr..

[9] Wahba S.A., El-shaheed A.A., Tawheed M.S., Mekkawy A.A. (2010). Osteoporosis knowledge, beliefs, and behaviours among Egyption female students. JASMR.

[10] Bollenbacher V.A. (2014). Effects of an Osteoporosis Educational Intervention: Knowledge and Self-Efficacy of Prevention in Young Adult Collegiate Females. https://scholar.valpo.edu/cgi/viewcontent.cgi?article=1056&context=ebpr.

[11] Hsieh C.H., Wang C.Y., McCubbin M., Zhang S., Inouye J. (2008). Factors influencing osteoporosis preventive behaviours: Testing a path model. J. Adv. Nurs..

[12] Sharma S.V., Hoelscher D.M., Kelder S.H., Diamond P., Day R.S. (2010). Hergenroeder, a psychosocial factors influencing calcium intake and bone quality in middle school girls. J. Am. Diet. Assoc..

[13] Piaseu N., Schepp K., Belza B. (2002). Causal analysis of exercise and calcium intake behaviours for osteoporosis prevention among young women in Thailand. Health Care Women Int..

[14] Tyler C.V., Werner J.J., Panaite V., Snyder S.M., Ford D.B., Conway J.L., Young C.W., Powell B.L., Smolak M.J., Zyzanski S.J. (2008). Barriers to supplemental calcium use among women in suburban family practice: A report from the cleveland clinic ambulatory research network (CleAR-eN). JABFM.

[15] Barzanji A.T., Alamri F.A., Mohamed A.G. (2013). Osteoporosis: A study of knowledge, attitude and practice among adults in Riyadh, Saudi Arabia. J. Community Health.

[16] WHO Health Topics: Adolescent Health. http://www.who.int/topics/adolescent_health/en/.

[17] Alamri F.A., Saeedi M.Y., Mohamed A., Barzanii A., Aldayel M., Ibrahim A.K. (2015). Knowledge, attitude, and practice of osteoporosis among Saudis. J. Egypt. Publ. Health Assoc..

[18] Al-Raddadi R., Bahijri S., Borai A., AlRaddadi Z. (2018). Prevalence of lifestyle practices that might affect bone health in relation to vitamin D status among female Saudi adolescents. Nutrition.

[19] Al-daghri N.M., Aljohani N., Al-attas O.S., Krishnaswamy S., Alfawaz H. (2015). Dairy products consumption and serum 25-hydroxyvitamin D level in Saudi children and adults. Int. J. Clin. Exp. Pathol..

[20] Ediriweera de Silva R.E., Haniffa M.R., Gunathillaka K.D.K., Atukorala I., Fernando E.D., Perera W.L. (2014). A descriptive study of knowledge, beliefs and practices regarding osteoporosis among female medical school entrants in Sri Lanka. Asia Pac. Fam. Med..

[21] Gammage K.L., Gasparotto J., Mack D.E., Klentrou P. (2011). Gender differences in osteoporosis health beliefs and knowledge and their relation to vigorous physical activity in university students. J. Am. Coll. Health.

[22] Shanthi Johnson C., McLeod W., Kennedy L., McLeod K. (2008). Osteoporosis health beliefs among younger and older men and women. Health Educ. Behav..

[23] Marcinow M.L., Randall Simpson J.A., Whiting S.J., Jung M.E., Buchholz A.C. (2017). Young adults’ perceptions of calcium intake and health: A qualitative study. Health Educ. Behav..

[24] Chouinard L.E., Simpson J.R., Buchholz A.C. (2012). Predictors of bone mineral density in a convenience sample of young Caucasian adults living in Southern Ontario. Appl. Physiol. Nutr. Metab..

[25] Puttapitakpong P., Chaikittisilpa S., Panyakhamlerd K., Nimnuan C., Jaisamrarn U., Taechakraichana N. (2014). Inter-correlation of knowledge, attitude, and osteoporosis preventive behaviours in women around the age of peak bone mass. BMC Women Health.

[26] Iwasaki E., Morakote N., Chaovistsaree S., Matsuo H. (2014). Bone mineral density and bone turnover among young women in Chiang Mai, Thailand. Kobe J. Med. Sci..

[27] Seo S., Chun S., Newell M.A., Yun M. (2015). Association between alcohol consumption and Korean young women’s bone health: A cross sectional study from the 2008 to 2011 Korea national health and nutrition examination survey. BMJ Open.

[28] Kim S., So W., Kim J., Sung D.J. (2016). Relationship between bone-specific physical activity scores and measures for body composition and bone mineral density in healthy young college women. PLoS ONE.

[29] Kim J., Jung M., Hong Y., Park J., Choi B. (2013). Physical activity in adolescence has a positive effect on bone mineral density in young men. J. Prev. Med. Publ. Health.

[30] Park D.-I., Choi-Kwon S., Han K. (2015). Health behaviours of Korean female nursing students in relation to obesity and osteoporosis. Nurs. Outlook.

[31] Amre H., Safadi R., Jarrah S., Al-Amer R., Froelicher E. (2008). Jordanian nursing students’ knowledge of osteoporosis. Int. J. Nurs. Pract..

[32] Al-Zu’bi A., Almuhtaseb N., Amayreh I. (2010). Osteoporosis awareness in a sample of teenage girls in Jordan. Jordan Med. J..

[33] Khan Y.H., Sarriff A., Khan A.H., Mallhi T.H. (2014). Knowledge, attitude and practice (KAP) survey of osteoporosis among students of a tertiary institution in Malaysia. J. Pharm. Res..

[34] Suriawati A.A., Majid H.A., Al-Sadat N., Mohamed M.N.A., Jalaludin M.Y. (2016). Vitamin D and calcium intakes, physical activity, and calcaneus bmc among school-going 13-year old Malaysian adolescents. Nutrients.

[35] Ford M.A., Bass M., Zhao Y., Bai J.-B., Zhao Y. (2011). Osteoporosis knowledge, self-efficacy, and beliefs among college students in the USA and China. J. Osteoporos..

[36] Mu M., Wang S., Sheng J., Zhao Y., Wang G., Liu K.Y., Hu C.L., Tao F.B., Wang H.L. (2014). Dietary patterns are associated with body mass index and bone mineral density in Chinese freshmen. J. Am. Coll. Nutr..

[37] Chen S.-W., Yang S.-C., Wang R.-H., Lin M.L. (2012). Osteoporosis prevention behaviours practiced among youth in Taichung City, Taiwan. Am. J. Health Behav..

[38] Sayed-Hassan R., Bashour H., Koudsi A. (2013). Osteoporosis knowledge and attitudes: A cross-sectional study among female nursing school students in Damascus. Arch. Osteoporos..

[39] Uddin R., Huda N.H., Jhanker Y.M., Jesmeen T., Imam M.Z., Akter S. (2013). Awareness regarding the Importance of Calcium and vitamin D among the undergraduate pharmacy students in Bangladesh. BMC Res. Notes.

[40] Sidor P., Głąbska D., Włodarek D. (2016). Analysis of the dietary factors contributing to the future osteoporosis risk in young Polish women. Natl. Inst. Publ. Health.

[41] Tonnesen R., Hovind P.H., Jensen L.T., Schwarz P. (2016). Determinants of vitamin D status in young adults: Influence of lifestyle, sociodemographic and anthropometric factors. BMC Public Health.

[42] Eleftheriou K.I., Rawal J.S., James L.E., Payne J.R., Loosemore M., Pennell D.J., Drenos F., Haddad F.S., Humphries S.E., Sanders J. (2013). Bone structure and geometry in young men: The influence of smoking, alcohol intake and physical activity. Bone.

[43] Ghaffari M., Nasirzadeh M., Rakhshanderou S.M.H.B., Harooni J. (2015). Osteoporosis-related knowledge among students of a medical sciences university in Iran: Calcium intake and physical activity. J. Med. Life.

[44] Bilal M., Haseeb A., Merchant A.Z., Rehman A., Arshad M.H., Malik M., Rehman A.H.U., Rani P., Farhan E., Rehman T.S. (2017). Knowledge, beliefs and practices regarding osteoporosis among female medical school entrants in Pakistan. Asia Pac. Fam. Med..

[45] Nguyen V.H., Wang Z. (2012). Osteoporosis knowledge of students in relevant healthcare academic programs. J. Osteoporos..

[46] Hervás G., Ruiz-Litago F., Irazusta J., Fernández-Atutxa A., Fraile-Bermúdez A.B., Zarrazquin I. (2018). Physical activity, physical fitness, body composition, and nutrition are associated with bone status in university students. Nutrients.

[47] Ito S., Ishida H., Uenishi K., Murakami K., Sasaki S. (2011). The relationship between habitual dietary phosphorus and calcium intake, and bone mineral density in young Japanese women: A cross-sectional study. Asia Pac. J. Clin. Nutr..

[48] Liberato S.C., Bressan J., Hills A.P. (2013). The role of physical activity and diet on bone mineral indices in young men: A cross-sectional study. J. Int. Soc. Sports Nutr..

[49] Chan M.F., Kwong W.S., Zang Y.L., Wan P.Y. (2007). Evaluation of an osteoporosis prevention education programme for young adults. J. Adv. Nurs..

[50] Du S., Mroz T.A., Zhai F., Popkin B.M. (2004). Rapid income growth adversely affects diet quality in China—Particularly for the poor!. Soc. Sci. Med..

[51] Heaney R.P., Abrams S., Dawson-Hughes B., Looker A., Marcus R., Matkovic V., Weaver C. (2000). Peak bone mass. Osteoporos. Int..

[52] Hammad L.F., Benajiba N. (2017). Lifestyle factors influencing bone health in young adult women in Saudi Arabia. Afr. Health Sci..

[53] Fehily A.M., Coles R.J., Evans W.D., Elwood P.C. (1992). Factors affecting bone density in young adults. Am. J. Clin. Nutr..

[54] Laitinen K., Valimaki M. (1991). Alcohol and bone. Calcif. Tissue Int..

[55] Maurel D.B., Boisseau N., Benhamou C.L., Jaffre C. (2012). Alcohol and bone: Review of does effects and mechanisms. Osteoporos. Int..

[56] Stránský M., Ryšavá L. (2009). Nutrition as prevention and treatment of osteoporosis. Physiol. Res..

[57] Holland A. (2017). Osteoporosis knowledge translation for young adults: New directions for prevention programs. Health Promot. Chronic Dis. Prev. Can..

[58] Schoenfeld R.E., Ng P., Henderson K., Wu S.-Y. (2010). Using the internet to educate adolescents about osteoporosis: Application of a tailored web-education system. Health Promot. Pract..

[59] Takahata Y. (2018). Usefulness of circuit training at home for improving bone mass and muscle mass while losing fat mass in undergraduate female students. Lipids Health Dis..

[60] Sanaeinasab H., Tavakoli R., Karimizarchi A., Amini Z.H., Farokhian A., Najarkolaei F.R. (2013). The effectiveness of education using the health belief model in preventing osteoporosis among female students. East. Mediterr. Health J..

[61] Zhang Y.-P., Li X.-M., Wang D.-L., Guo X.-Y., Guo X. (2012). Evaluation of educational program on osteoporosis awareness and prevention among nurse students in China. Nurs. Health Sci..

[62] Bohaty K., Rocole H., Wehling K., Waltman N. (2008). Testing the effectiveness of an educational intervention to increase dietary intake of calcium and vitamin D in young adult women. J. Am. Acad. Nurse Pract..

[63] Goodman S., Morrongiello B., Meckling K. (2016). A randomized, controlled trial evaluating the efficacy of an online intervention targeting vitamin D intake, knowledge and status among young adults. Int. J. Behav. Nutr. Phys. Act..

[64] Ha E., Caine-bish N., Holloman C., Lowry-gordon K. (2009). Evaluation of effectiveness of class-based nutrition intervention on changes in soft drink and milk consumption among young adults. Nutr. J..

